# Intermittent levosimendan infusion in ambulatory patients with end-stage heart failure: a systematic review and meta-analysis of 984 patients

**DOI:** 10.1007/s10741-021-10101-0

**Published:** 2021-04-11

**Authors:** Hagar Elsherbini, Osama Soliman, Casper Zijderhand, Mattie Lenzen, Sanne E. Hoeks, Rasha Kaddoura, Mohamed Izham, Abdulaziz Alkhulaifi, Amr S. Omar, Kadir Caliskan

**Affiliations:** 1 Department of Cardiology, Erasmus University Medical Centre, Rotterdam, Netherlands; 2 Utrecht University of Applied Sciences, Utrecht, Netherlands; 3 Department of Cardiology, National University of Ireland, Galway, Ireland; 4 Department of Clinical Pharmacy, Hamad Medical Corporation, Doha, Qatar; 5 College of Pharmacy, QU Health, Qatar University, Doha, Qatar; 6 Department of Cardiothoracic Surgery/Cardiac Anaesthesia & ICU, Heart Hospital, Hamad Medical Corporation, Doha, Qatar; 7 Department of Critical Care Medicine, Beni Suef University, Beni Suef, Egypt; 8 Department of Clinical Medicine, Weill Cornell Medical College, Doha, Qatar

**Keywords:** Heart failure, Levosimendan, Survival, Functional capacity, Safety, Efficacy, Ambulatory

## Abstract

We sought to synthesize the available evidence regarding safety and efficacy of intermittent levosimendan (LEVO) infusions in ambulatory patients with end-stage heart failure (HF). Safety and efficacy of ambulatory intermittent LEVO infusion in patients with end-stage HF are yet not established. We systematically searched MEDLINE, EMBASE, SCOPUS, Web of Science, and Cochrane databases, from inception to January 30, 2021 for studies reporting outcome of adult ambulatory patients with end-stage HF treated with intermittent LEVO infusion. Fifteen studies (8 randomized and 7 observational) comprised 984 patients (LEVO [*N* = 727] and controls [*N* = 257]) met the inclusion criteria. LEVO was associated with improved New York Heart Association (NYHA) functional class (weighted mean difference [WMD] −1.04, 95%CI: −1.70 to −0.38, *p* < 0.001, 5 studies, *I*^2^ = 93%), improved left ventricular (LV) ejection fraction (WMD 4.0%, 95%CI: 2.8% to 5.3%, *p* < 0.001, 6 studies, *I*^2^ = 9%), and reduced BNP levels (WMD −549 pg/mL, 95%CI −866 to −233, *p* < 0001, 3 studies, *I*^2^ = 66%). All-cause death was not different (RR 0.65, 95%CI: 0.38 to 1.093, *p* = 0.10, 6 studies, *I*^2^ = 0), but cardiovascular death was lower on LEVO (RR 0.34, 95%CI: 0.13 to 0.87, *p* = 0.02, 3 studies, *I*^2^ = 0) compared to controls. Furthermore, health-related quality of life (HRQoL) was improved alongside with reduced LV size following LEVO infusions. Major adverse events were not different between LEVO and placebo. In conclusion, intermittent LEVO infusions in ambulatory patients with end-stage HF is associated with less frequent cardiovascular death alongside with improved NYHA class, quality of life, BNP levels, and LV function. However, the current evidence is limited by heterogeneous and relatively small studies.

**Supplementary information:**

The online version contains supplementary material available at 10.1007/s10741-021-10101-0.

## Introduction

Chronic heart failure (HF) is a major cause of recurrent hospitalizations and mortality. Various estimates revealed that 1–2% of the world population in developed countries are living with HF and the prevalence is increasing [1]. Most patients suffer from symptoms that limit their daily life activities and consequently lead to poor quality of life. In addition, patients with HF often require frequent hospitalizations, which is more pronounced than in many other chronic conditions. Therefore, healthcare costs for the care of HF patients are high and rising [2, 3]. More importantly, it is estimated that more than half of the patients with chronic HF die within 5 years after their diagnosis [3]. In selected patients with end-stage HF in whom direct left ventricular assist device implantation (LVAD) or heart transplantation (HTx) is not achievable, palliative ambulatory inotropic infusions could be considered. Furthermore, in countries with extreme long waiting for a donor heart, it may be used as a bridge-to-transplantation if an LVAD implantation is not feasible or not available [4]. This inotropic support includes dopamine, dobutamine, milrinone, enoximone, and in some countries levosimendan (LEVO). Repetitive and continuous administration of conventional inotropes such as milrinone and dobutamine could provide hemodynamic relief in patients with end-stage HF and is associated with symptomatic improvements. Yet, no positive effect on repeated hospitalizations has been observed with those conventional inotropes and their chronic use is associated with a higher mortality risk, possibly due to toxic adverse effects. In contrast to conventional inotropes, LEVO is a new inotropic agent that acts as a calcium sensitizer. It sensitizes troponin C without increasing intracellular calcium concentration or exacerbating ischemia, and as an inodilator, it reduces the cardiac pre-, and afterload. LEVO acts as a potassium channel opener, which has an active metabolite (OR1896) that peaks approximately 80 to 90 h after administration and is associated with hemodynamic improvements that are sustained for a week [5]. The advantages of LEVO include beneficial symptomatic, hemodynamic, and neurohormonal effects, and improved peripheral organ perfusion and renal function. Importantly, there is no effect attenuation in patients using beta-blockers [2], which is currently one of the main HF treatment agents. In early studies, LEVO has shown to decrease mortality [6], improve hemodynamics, and reduce symptoms. However, the occurrence of hypotension and arrhythmia was increased. Furthermore, its impact on HF hospitalization and mortality is not consistent [7–9]. Therefore, we conducted a systematic review and meta-analysis of the current literature to synthesize evidence regarding exploring the efficacy and the safety of LEVO on different outcomes in ambulatory end-stage HF patients receiving intermittent LEVO infusions.

## Methods

### Search strategy

This systematic review was performed and reported according to the Preferred Reporting Items for Systematic Reviews and Meta-Analyses (PRISMA) guidelines [10]. From inception to September 27, 2019, all relevant items were identified in collaboration with a Librarian in the Erasmus University Medical Centre. We searched Embase, Medline Ovid, Web of Science, Cochrane CENTRAL register of trials, and Google Scholar for articles published until the date of search. The full search is available in the Supplementary Material Appendix 1. We updated our search using same methodology on January 30, 2021, which resulted in 48 studies. However, none of those studies were eligible to be included in this systematic review and meta-analysis.

### Inclusion and exclusion criteria

Adult (≥18 years) ambulatory patients with end-stage HF receiving intermittent intravenous LEVO infusions were included. We included all clinical studies (e.g., randomized trial, observational cohort, case-control, case-series) containing ≥10 patients and published in the last 30 years. Case reports, editorials, reviews, studies included hospitalized patients or orally administered LEVO, and articles that are not in English language were excluded.

### Study selection

Two researchers (H.E. and O.S.) independently reviewed abstracts and full texts in an unblinded standardized manner. Disagreements between the researchers to include a study were discussed and resolved before final approval. Furthermore, references in selected articles were independently cross-checked by the 2 researchers for other relevant studies.

### Data extraction

Two authors (H.E. and O.S.) extracted the data independently, using a pre-defined standardized data extraction form. This extracted data was compared and confirmed with the original articles. Data extraction included study characteristics (e.g., author and study design), number of patients (categorized as receiving LEVO or controls), study duration, and treatment characteristics (LEVO dose and method of administration). Furthermore, patient demographics (e.g., age and sex), baseline characteristics (e.g., ischemic HF etiology), and outcomes (primary endpoints and secondary endpoints) were documented. The following endpoints were extracted if available: mortality, hospitalization, length of stay in hospital, New York Heart Association (NYHA) class, 6-Minute Walk Distance (6MWD), Kansas City Cardiomyopathy Questionnaire(KCCQ), brain natriuretic peptide (BNP), N-terminal pro-BNP (NT-pro-BNP), left ventricular (LV) ejection fraction (LVEF), mean arterial pressure (MAP), LV end-systolic (LVES), and LV end-diastolic (LVED) wall stress. Other outcome variables that included urgent or elective HTx and LVAD implantation were collected when available. In addition, reported complications or side effects of the use of inotropic agents, safety aspects of LEVO including tachycardia, atrial fibrillation, ventricular arrhythmias, and hemodynamic instability were collected. Microsoft Office Excel was used for data extraction.

## Risk of bias assessment, quality, and validity of included studies

The risk of bias and quality of the included studies were assessed by the two independent reviewers (H.E. and O.S.) including the use of Newcastle-Ottawa scale (NOS) [11]. All relevant discrepancies were resolved by discussion until consensus achieved between the two reviewers.

## Statistical analysis

Random and fixed effects models using the Der Simonian and Laird method were used to pool the outcomes. Continuous paired data was presented as mean and standard deviation when applicable. Dichotomous unpaired data was presented as mean difference and risk ratio. A *p* value <0.05 was considered statistically significant and a 95% confidence interval (95%CI) was calculated. The Cochrane Q statistic and inconsistency factor (*I*^2^) were used to assess heterogeneity. *I*^2^ value above 50% was considered as significant heterogeneity. Egger’s test was used to assess the risk of publication bias [12]. Comprehensive Meta-Analysis (CMA) v2.2.064 (Biostat, Englewood, NY, USA) was used to calculate the pooled outcomes and to generate forest plots.

## Results

**Fig. 1:**
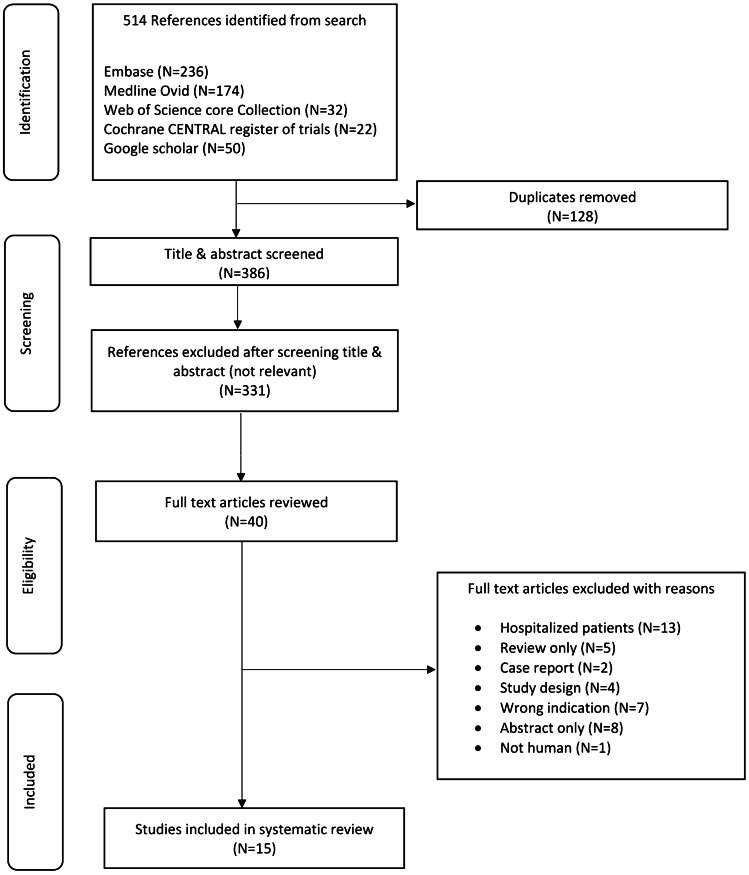
PRISMA flowchart of selection of studies for inclusion

**Fig. 2:**
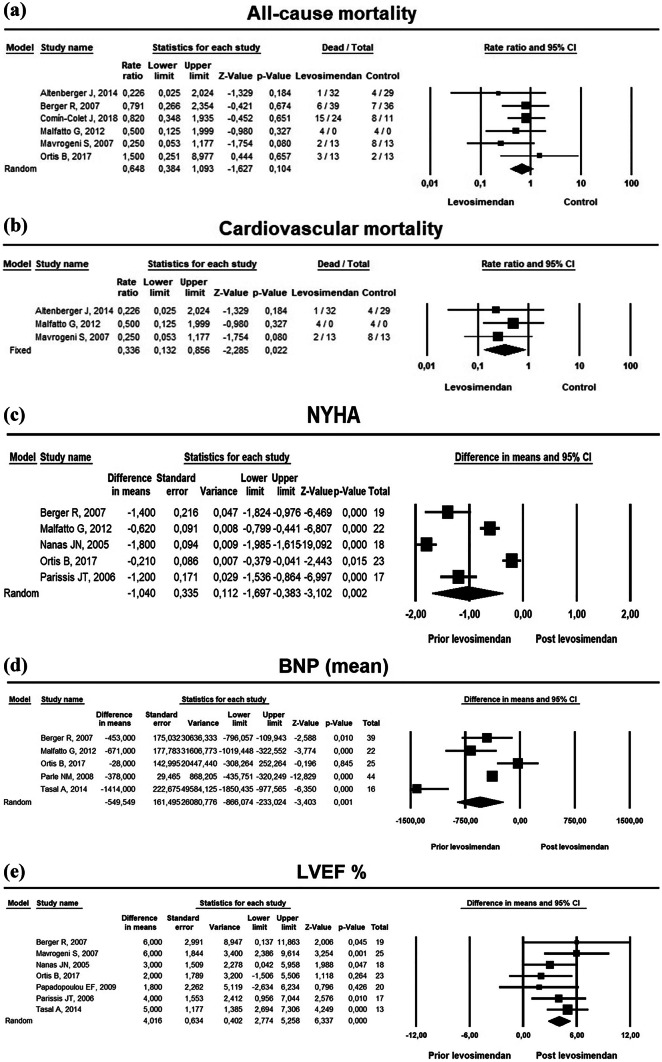
Central Illustration. Meta-analysis of effects of Levosimendan infusion versus baseline on risk of all-cause death (**a**); risk of cardiovascular mortality (**b**); changes in New York Heart Failure Association (NYHA) functional class (**c**); changes in brain natriuretic peptide (BNP, **d**); and changes in left ventricular ejection fraction (LVEF, **e**)

The search strategy resulted in 514 studies. After removal of duplicates, 386 studies remained. After reviewing the title and abstract, another 331 studies were removed due to irrelevance. Of the remaining 55 studies, 15 studies [6–9, 12–22] met the predefined inclusion criteria and were consequently included in this review. Figure 1 displays the PRISMA flowchart. This systematic review included 984 patients in 8 randomized controlled [6–9, 13, 14, 16, 20] and 6 non-randomized [12, 15, 17–19, 21, 22] studies (Fig. 2)*.* Study characteristics are described in Tables 1, 2, 3, and Supplementary (Tables 1, 2A, and 2B).

### Efficacy of LEVO

One study [17] reported that the indication for LEVO was palliation in 116 (63%) patients and as a bridge in 69 (47%) patients. Of the latter, LEVO was used as a bridge to HTx in 33 (48%) patients, a bridge to candidacy to HTx or a bridge to LVAD in 28 (41%) patients, and to as a bridge to decision to further options in 8 (12%) patients.

### Survival after LEVO

Nine studies [6–9, 12–14, 18, 22] reported survival following LEVO (Table 4 and Fig. 2). Three studies [6, 12, 22] were not included in the meta-analysis due to a lack of a comparator arm [22] and due to a combined dobutamine plus LEVO in the main arm [6, 12]. A random effect model meta-analysis of 6 studies [7–9, 13, 14, 18] showed no difference in all-cause death between LEVO (*n* = 222) and controls (*n* = 175) with a weighted mean relative risk (RR) of 0.65 (95%CI: 0.38 to 1.09, *p* = 0.104, *I*^2^ = 0). In contrast, fixed effect model meta-analysis of 3 studies [7, 13, 14] showed a reduction in cardiovascular death on LEVO (*n* = 110) compared to controls (*n* = 93) with a weighted mean RR of 0.336 (95% CI: 0.132 to 0.856, *p* = 0.022, *I*^2^ = 0).

**Table 1:** Metadata of the 15 studies included in the systematic review and meta-analysis

Author	Date of Publication	Journal	Acronym	Database
Altenberger et al.	2014	European Journal of Heart Failure	LevoRep	Embase
Berger et al.	2006	The European Journal of Heart Failure	NA	Embase
Bonios et al.	2011	International Journal of Cardiology	NA	Embase
Comín-Colet et al.	2018	European Journal of Heart Failure	LION-HEART	Embase
Drakos et al.	2008	Journal of Cardiovascular Pharmacology	NA	Embase
Malfatto et al.	2012	Journal of Cardiovascular Pharmacology	NA	Embase
Mavrogeni et al.	2007	Journal of Cardiac Failure	NA	Embase
Najjar et al.	2018	ESC Heart Failure	NA	Embase
Nanas et al.	2015	The American Journal of Cardiology	NA	Embase
Oliva et al.	2018	International Journal of Cardiology	NA	Embase
Ortis et al.	2016	Journal of International Medical Research	NA	Embase
Papadopoulou et al.	2009	Hellenic J Cardiol	NA	Embase
Parissis et al.	2006	Cardiovascular Medicine	NA	Embase
Parle et al.	2008	Heart, Lung and Circulation	NA	Embase
Tasal et al.	2014	Medical Science Monitor	NA	Embase

*NA* not available

**Table 2:** Methodology of the 15 studies included in the systematic review—study design, demographics, and comparator arm

Author	Publication year	Study design	No of patients	(%male)	Ischemic etiology	Comparator	Study duration (months)	Interim analysis
Altenberger et al.	2014	RCT	120	79%	62%	Placebo	6	2
Berger et al.	2006	RCT	75	82%	NR	PGE1	12	3
Bonios et al.	2011	RCT	63	NR	NR	Dobutamine	6	
Comín-Colet et al.	2018	RCT	69	NR	NR	Placebo	6	3 (1ry)
Drakos et al.^a^	2008	CS	162	93%	54%	OMT	6	
Malfatto et al.	2012	RCT	33	73%	77%	Furosemide	12	4
Mavrogeni et al.	2007	RCT	50	80%	52%	SOC	6	
Najjar et al.	2018	CS	23	83%	NR	No comparator	24Hrs	
Nanas et al.	2015	RCT	36	94%	NR	Dobutamine	3	
Oliva et al.	2018	PR	185	80%	60%	No comparator	6	
Ortis et al.	2016	CCS	50	72%	76%	No LEVO	12	6
Papadopoulou et al.	2009	CS	20	90%	80%	No comparator	6	
Parissis et al.	2006	RCT	25	92%	84%	Placebo	4^a^	
Parle et al.	2008	CS	44	NR	48%	No comparator	48	
Tasal et al.	2014	CS	29	69%	NR	#No comparator	6	

*RCT* randomized control trial, *CS* cohort study, *CCS* cross sectional, *PGE1* prostaglandin E1, *NR* not reported, *LEVO* levosimendan, *OMT* optimal medical therapy, *SOC* standard of care

^a^114 days

**Table 3:** Methodology of the 15 studies included in the systematic review—infusion protocol

Author	Study duration (months)	No of cycles	Frequency of infusions	Duration of infusion	Levosimendan loading dose (LD)	Levosimendan maintenance dose (MD)
Altenberger et al.	6 Months	4	Bi-monthly	6 h	No loading dose	0.2 µg/kg/min
Berger et al.	12 Months	3	Monthly	24 h	if SBP ≥ 95 mmHg, a LD of 12 μg/kg for 10 min	Rate of 0.1 μg/kg/min for 24 h
Bonios et al.	6 Months	24	Weekly	6-h	No loading dose	0.3 µg/kg/min LEVO & DOB 10 mg/kg/min + LEVO 0.2 mg/kg/min
Comín-Colet et al.	3 Months: 1ry 6 Months: 2nry	6	Bi-monthly	6-h	No loading dose	0.2 µg/kg/min
Drakos et al.	6 Months	24	Weekly	8-h	No loading dose	10 µg/kg/min DOB or 0.3 µg/kg/min LEVO, or both
Malfatto et al.	12 Months	4	Monthly	24-h	No loading dose	0.1 to 0.4 mg/kg/min (max 12.5 mg per session)
Mavrogeni et al.	6 Months	6	Monthly	24-h	No loading dose	0.2 µg/kg/min
Najjar et al.	24 h	1	-	24-h	No loading dose	0.1 µg/kg/min
Nanas et al.	3 M	6	Bi-Monthly	24-h	First 24 h DOB infusion, then a 6 mg/kg IV bolus of LEVO	6 mg/kg bolus LEVO IV, followed by a 24-h infusion of 0.2 µg/kg/min
Oliva et al.	6 Months	6	Monthly	24-h	No loading dose	0.05 to 0.2 µg/kg/min
Ortis et al.	12 Months	12	Monthly	24-to 48-h	No loading dose	0.1—0.2 µg/kg/min (max 12.5 mg); cycle of 3 infusions (12.5 mg) for 24–48 hrs
Papadopoulou et al.	6 Months	6	Monthly	24-h	No loading dose	0.1 mg/kg/min
Parissis et al.	4^a^ Months	5	3 weeks	24-h	First 10-min IV bolus of 6 µg/kg	5 cycles of 24 h infusion at 0.1 µg/kg/min
Parle et al.	48 Months	2–26	Mean 66.2 (12.0) days	24 h	First 10-min IV bolus of 6–12 µg/kg	a 24 h of 0.1 µg/kg/min uptitrated hourly (max 0.4 µg/kg/min)
Tasal et al.	6 Months	3 vs 1	Single vs monthly	24 h	First 10-min IV bolus of 6 µg /kg	Main rate of 0.1 µg/kg/min

For abbreviations, see Table 1

^a^114 days

### Number of hospitalizations and length of hospital stay

LEVO was associated with a reduction in the number of hospitalizations in 3 studies [9, 17, 18]. Compared to the 6-month before randomization, the number of hospitalizations was reduced on LEVO while increased on placebo, during 6 and 12 months [18]. Comin-Colet et al. reported improved all-, HF-, and cardiovascular-related hospitalization during 6 months following LEVO [9] compared to before LEVO use. LEVO was associated with a mean decrease in the length of hospital stay, compared to the 6-month before therapy, by 4.2 days [17] (*p* = 0.0001) and 7.4 days (*p* < 0.05) [18], respectively. In one study [18], compared with the 6-month before treatment, a mean decrease in the length of hospital stay by 0.3 days was seen during 12 months following LEVO compared with 13.4-day mean increase in controls (*p* < 0.05).

### Functional status and quality of life following LEVO

Changes in NYHA functional class following LEVO were reported in 8 studies [8, 9, 12, 13, 18, 20–22] compared with baseline or compared with single LEVO dose. LEVO was also compared to placebo [9], furosemide [13], and to standard HF therapy [18]. Random-effect model meta-analysis of 5 out of 8 studies [8, 13, 16, 18, 20] showed NYHA class improvement following LEVO use compared to before use, with a weighted mean difference (WMD) of −1.04 (95%CI: −1.6 to −0.4, *p* < 0.001). However, heterogeneity between studies was high, inconsistency index (*I*^2^ = 98% for studies with 3- to 12-month follow-up). NYHA functional status was significantly improved by 0.1 to 1.4 classes following LEVO in 6 studies over 3 to 12 months. In one study, no significant change in NYHA functional class was seen on LEVO, although NYHA functional class was worsened in controls [18]. Only one study [7] reported no statistical difference in improvements of 6MWD or KCCQ at 8 and 24 weeks in patients receiving LEVO versus placebo. Two studies [9, 19] reported HRQoL assessments on LEVO. Comin-Colet et al. [9] found that LEVO was associated with less often clinically significant (reduction of 5 points on the EQ-5D visual analog scale) decline in HRQoL [5/24 LEVO patients (21%) versus 7/11 placebo patients (63%), *p* = 0.02] [9]. Papadopoulou et al. reported that the Specific Activity Questionnaire (SAQ), Left Ventricular Dysfunction Questionnaire (LVD-36), and the Minnesota Living with Heart Failure Questionnaire (MLHFQ) showed a significant improvement in HRQoL during 6 months following LEVO [19].

### Laboratory parameters

Laboratory parameters associated with LEVO use mainly included BNP, NT-pro-BNP, serum creatinine, and estimated glomerular filtration rate (eGFR). Other laboratory indices included troponin T, IL-6, and tumor necrosing factor-alpha (TNF-α).

### BNP and NT-pro-BNP

Baseline BNP versus post-LEVO infusion was reported in five studies [8, 13, 18, 21, 22] and was included in the meta-analysis. Random-effect model meta-analysis showed a significant decrease in BNP levels post-LEVO with a WMD of −550(95%CI: −866 to −233, *p* < 0.001), regardless of the follow-up duration (range 3 to 48 months) compared to pre-LEVO. The heterogeneity between the studies was very high (*I*^2^ = 87%). NT-pro-BNP was reported in four studies [7, 9, 15, 20]. NT-pro-BNP was compared at post-LEVO follow-up versus baseline [*n* = 2] over 12 weeks and 24 weeks [15, 20] and two studies compared NT-pro-BNP in patients receiving LEVO versus placebo over 3 months [7, 9]. NT-pro-BNP was significantly decreased (range 966 to 2530 pg/mL) after LEVO in all four studies. Detailed laboratory findings are presented in Supplementary Tables 3A and 3B.

### Renal function following LEVO

Changes in serum creatinine following LEVO were reported in 6 studies [6, 12, 16, 18, 20, 21]. No change in serum creatinine levels was found (148.8 [6.6] μmol/L pre-infusion versus 145 [6.9] μmol/L post-infusion [21] and 115 μmol/L at baseline versus 115 μmol/L at final infusion [20]. Likewise, no significant change was seen at 6 months on LEVO (1.8 ± 0.7 mg/dL vs 1.6 ± 1 mg/dL, *p* = 0.056) compared with baseline [12]. In Bonios et al. [6] study, there was no significant change in serum creatinine at 3 months compared to baseline among patients receiving LEVO versus dobutamine plus LEVO and versus patients receiving only dobutamine. Nanas et al. [16] found that serum creatinine did not significantly change among 45-day survivors on LEVO compared to baseline. In only one study [18], serum creatinine was worsened (2.0 ± 0.1 versus 1.7 ± 0.7 mg/dL, *p* < 0.05) on LEVO, compared with baseline, but was stable in controls. In 3 studies [13, 15, 18], eGFR was compared at post-LEVO versus pre-LEVO [15], versus guideline-directed medical therapy [18], and versus furosemide [13]. Najjar et al. found that eGFR insignificantly changed from 62 to 61 mL/min/m^2^ (*p* = 0.96) following LEVO [15]. Malfatto et al. [13] found that compared to baseline, eGFR was insignificantly changed following first LEVO infusion cycle (46 ± 8 to 49 ± 9, *p* = 0.12) similar to controls (45 ± 10 to 43 ± 11, *p* = NS). Likewise, eGFR was insignificantly changed following 4 monthly cycles of LEVO (46 ± 8 to 47 ± 9, *p* = NS) similar to controls (45 ± 10 to 44 ± 8, *p* = NS). Ortis et al. found that 12-month eGFR worsened compared with baseline values in the LEVO group (45.6 ± 19.4 vs 39.7 ± 16.3, *p* < 0.05), but 12-month values did not differ from controls (42.9 ± 32.4 vs 38.8 ± 30.4, *p* = NS), respectively [18].

### Other laboratory markers

Parissis et al. [20] found no difference in the number of cases with a positive troponin T (>0.01 ng/mL but not exceeding 0.1 ng/mL) in most cases in the LEVO versus placebo at baseline; however, it was higher at 6 months in the placebo group (*p* < 0.05). In addition, two studies [20, 22] presented changes in inflammatory markers following LEVO. Parissis et al. [20] found that at 6 months, interleukin-6 was decreased (13.1 [3.8] pg/mL baseline versus 10.8 [7.2] pg/mL final) on LEVO. Another study [22] also found significant reduction in inflammatory markers, specifically interleukin-6 and TNF-α at 6 months following repeated dose LEVO compared with baseline. In contrast, worsening in interleukin-6 in patients who received a single-dose LEVO was seen at 6 months (Supplementary Table 3B).

### Echocardiographic parameters

Echocardiographic parameters following LEVO included LV ejection fraction (LVEF), LV end-diastolic volume, LV end-systolic volume, LV end-systolic wall stress, LV fractional shortening, and MR severity.

#### LV size and function

Changes in LVEF were seen in 7 studies [8, 14, 16, 18–20, 22] following LEVO. LVEF was compared in patients receiving LEVO versus controls during 6- [14] and 12-month [8, 18] follow-up, and at 6 months versus baseline [19, 20]. LVEF was compared in patients receiving single versus repeated dose of LEVO at 6 months [22] and at 45 days following LEVO versus baseline among survivors [16]. In all studies, LVEF was increased (range 2% to 6%) following LEVO Supplementary Tables 3A and 3B*.* Regardless of the follow-up duration, random-effect model meta-analysis showed a mean significant improvement in LVEF post-LEVO (*n* = 135) by a WMD of 4.0% (95%CI: 2.7% to 5.3%, *p* < 0.001), compared with baseline. Furthermore, the heterogeneity among the studies was very low (*I*^2^ = 0). Changes in LVEDV and LVESV following LEVO use were reported in 3 studies [14, 20, 22]. Two studies [14, 20] found that LVEDV and LVESV were decreased (range −13–18 mL/m^2^) at 6 months on LEVO while it was increased in controls [14, 20], compared with baseline. LVEDV and LVESV were reduced at 6 months following repeated dose LEVO while increased following single-dose LEVO, compared with baseline [22]. However, only one study [18] found that LVEDV was reduced at 12 months on LEVO, while it was increased in controls (Supplementary Tables 3A and 3B)*.* LVES wall stress was reduced (change −111 g/cm^2^ [*p* < 0.05]) on LEVO in one study [20]. Likewise, in only one study [14], LVFS was increased and MR grade was decreased at 6 months on LEVO compared to controls (both *p* < 0.05, Supplementary Table 3B).

### Cost difference between LEVO use versus no LEVO

Oliva et al. [17] is the only study, which addressed the cost-effectiveness of LEVO infusion in ambulatory patients with end-stage HF. Cost per patient, defined as the daily cost multiplied by total days, during 6-month use of LEVO was €5616 [range €4128 to €8215] versus €7290 [range €2551 to €11164] during the 6-month before treatment (*p* = 0.05) with a €1157 (€8676) of costs saving.

### Safety of LEVO

Adverse events of intermittent LEVO infusions were reported in 10 studies. Hypotension was seen in 2.2% to 36% of patients. The frequency of hypotension was not different between LEVO vs PGE1 [8] or vs placebo [9]. Asymptomatic non-sustained ventricular tachycardia was observed during 4 (2.5%) infusions [21], ventricular arrhythmias in 16 (8.6%) [17], and atrial fibrillation in 1 (0.5%) [17]. Arrhythmia rates were similar between LEVO and placebo in 3 studies [7, 13, 14] and versus furosemide [13]. Detailed LEVO safety is presented in Supplementary file and Supplementary Table 4.

### Risk of bias assessment

As shown in Tables 5 and 6, 10 studies [6, 7, 9, 16–19, 21] have poor quality, and 5 studies [8, 13, 14, 20, 22] have good quality on the Ottawa-Newcastle scale. Furthermore, heterogeneity among the studies included in the meta-analysis was low for all-cause mortality, cardiovascular death, and LVEF. In contrast, high heterogeneity was seen in studies reporting on NYHA and BNP (Table 6).

**Table 4:** Summary of events and key characteristics of the 9 studies who reported mortality following levosimendan intermittent infusions

Author	No of patients	Comparator	Study duration (months)	Mortality reported	LEV-events	LEVO-total	Control-events	Control-total	Survival OR	Survival CI	Survival *p* value
Included in the meta-analysis											
Altenberger et al.	120	Placebo	6	Yes	1	63	4	57	0.21	0.02–1.97	NS
Berger et al.	75	PGE1	12	Yes	6	39	7	36	0.75	0.23–2.51	NS
Comín-Colet et al.	69	Placebo	6	Yes	14	48	7	21	0.82	0.27–2.47	NS
Malfatto et al.	33	Furosemide	12	Yes	4	22	4	11	0.39	0.08–2.00	NS
Mavrogeni et al.	50	SOC	6	Yes	2	25	8	25	0.18	0.03–0.98	< 0.05
Ortis et al.	50	No LEVO	12	Yes	3	25	2	25	1.5	NR	NS
Not included in the meta-analysis											
Bonios et al.	63	Dobutamine	6	Yes	14	42	8	21	0.81	0.27–2.42	NS
Drakos et al.^a^	162	OMT	6	Yes	19	29	20	22	0.72	NR	*NR
Tasal et al.	29	None^b^	6	Yes	0	29	0	NR	NR	NR	NR

*CS* cross sectional, *OMT* optimal medical therapy, *SOC* standard of care, *NR* not reported or not relevant, *RCT* randomized control trial

^*^p < 0.001 for inotropic vs SOC)

^a^in Drakos et al., Intravenous inotropic versus standard of care were compared. The inotropic patients comprised 3 subgroups (Levosimendan alone, Dobutamine alone, and a combination of both)

^b^Patients on repeated vs single dose Levosimendan were compared

**Table 5:** Risk of bias assessment of studies included in the in the systematic review and meta-analysis

Meta-data		Methodology				Outcomes LEVO vs control		Newcastle-Ottawa scale			
Author (year)	Publication date	Study design	Cohorts comparability at baseline	Renal function comparability at baseline	Adequately powered	Survival	Hospitalization	Selection	Comparability	Outcome	Quality
Altenberger et al.	2014	+ +	+	-	(63 vs 57)	↑	NR	****	*	*	Poor
Berger et al.	2006	+ -	+	NR	(39 vs 36)	=	NR	****	*	**	Good
Bonios et al.	2011	+ -	+	+	(21 vs 21 vs 21)	↑	NR	****	*	*	Poor
Comín-Colet et al.	2018	+ +	+	NR	(48 vs 21)	=	↑	****	**	*	Poor
Drakos et al.	2008	+ -	-	NR	(140 vs 22)	↑	NR	****	-	*	Poor
Malfatto et al.	2012	+ -	+	+	(22 vs 11)	↑	NR	****	*	**	Good
Mavrogeni et al.	2007	+ -	+	NR	(25 vs 25)	NR	NR	****	*	***	Good
Najjar et al.	2018	+ -	NA	+	−23	NR	NR	****	-	-	Poor
Nanas et al.	2015	+ -	-	+	(18 vs 18)	NR	NR	****	-	*	Poor
Oliva et al.	2018	- +	NA	NR	−185	NR	↑	****	-	**	Poor
Ortis et al.	2016	–	+	+	(25 vs 25)	=	NR	****	*	*	Poor
Papadopoulou et al.	2009	+ -	NA	NR	−20	NR	NR	****	-	*	Poor
Parissis et al.	2006	+ -	+	+	(17 vs 8)	NR	NR	****	*	***	Good
Parle et al.	2008	+ -	+	+	−44	NR	NR	****	*	*	Poor
Tasal et al.	2014	+ -	+	NR	(16 vs 13)	↑	NR	****	*	***	Good

Study design: prospective (+); retrospective (-); single centre (-), multicentre (+)

Cohort comparability at baseline: (+) means comparable; NA = not applicable; (-) is not comparable

*NA* not available, *NR* not reported

**Table 6:** Heterogeneity between studies included in the meta-analysis regarding different outcome variables

Outcome	*Q* value	*df* (*Q*)	*P* value	*I* squared	Heterogeneity
All-cause mortality	3,736	5	0,588	0,000	Low
Cardiovascular mortality	0,581	2	0,748	0,000	Low
NYHA (3–12 m)	173,328	4	0,000	97,692	High
BNP regardless follow-up (6–48 m)	30,350	4	0,000	86,820	High
LVEF regardless follow-up duration (3–12 months)	4,980	6	0,546	0,000	Low

*NYHA* New York Heart Failure Association, *LVEF* left ventricular ejection fraction, *BNP* brain natriuretic peptide

## Discussion

To the best of our knowledge, this systematic review is the largest (*N* = 984) to date to assess the efficacy and safety of intermittent LEVO infusions in ambulatory patients with end-stage HF. There are several important findings. First, intermittent LEVO infusions were associated with improved functional capacity, quality of life, LV size, and function. Second, rates of all-cause death were not statistically different between patients who received LEVO versus placebo. However, intermittent LEVO infusions were associated with lower rates of cardiovascular death compared to placebo, furosemide, or to standard of care. Furthermore, evidence of LEVO efficacy, while promising, is yet based on a small, heterogeneous studies, with conflicting finding among some endpoints. Therefore, it must be confirmed in a larger prospective randomized study. Finally, intermittent LEVO infusions in ambulatory patients with end-stage HF were safe with only few episodes of mostly non-dose limiting systemic arterial hypotension and arrhythmias. But the frequency of these side effects was not statistically higher than the placebo. Patients with end-stage HF often require the administration of inotropic agents, via continuous or intermittent infusion. This method is typically used in hospitalized patients with end-stage HF aiming at improving quality of life and hopefully keeping patients alive until successful bridging to LVAD or to HTx [3]. Conventional inotropes such as dobutamine, dopamine, and norepinephrine can immediately improve clinical status and correct hemodynamic instability, at the expense of further myocardial damage via increased myocardial oxygen requirements, cardiotoxicity, and pro-arrhythmic effects. These side effects might significantly limit their usefulness [3, 23, 24]. Sometimes, continuous home-based inotropic support could be initiated for pure palliative reasons as a part of end-of life support [4]. In contrast, LEVO firstly registered in 2000 in Sweden and thereafter in several European countries is non-conventional inotropic agent, which act as a calcium sensitizer and inodilator with a unique mode of action that has been developed for treatment of decompensated HF. It increases cardiac contractility without increasing myocardial oxygen demand or exacerbating ischemia. Other factors favoring LEVO, as a positive inotropic, include the lack of any attenuation of effect in patients treated with beta-blockers alongside with an extended duration of action. Furthermore, LEVO also acts like a vasodilator, by opening adenosine tri-phosphate (ATP)-sensitive potassium channels in vascular muscles, which results in muscle relaxation [5]. It reduces peripheral vascular resistance, improves diastolic function, and therefore the pre-and afterload of the failing heart.

### Efficacy of LEVO in this systematic review

The intermittent administration of LEVO in ambulatory patients with end-stage HF is consistently associated with improved echocardiographic parameters, reverse LV remodeling, lower filling pressures, and lower biomarkers of LV failure. This is reflected by the improvement in HRQoL alongside with significant reduction in the number and length of hospitalizations, consequently lower cost of HF care at 6 and 12 months in several studies. The improved functional status and reduced hospitalizations following LEVO could be explained by halted HF disease progression as seen in the reduction of BNP levels following LEVO infusion. In contrast, patients who did not receive LEVO showed HF progression, as reflected by a significant increase in BNP levels during similar follow-up. However, reduction in BNP was not consistent among all studies that reported BNP level following LEVO. In only one study, the 6MWD distance and KCCQ were improved on LEVO, but the change was not statistically different from placebo, probably due to small sample size [7]. Importantly, the use of LEVO was associated with a numerically, but statistically insignificant, lower rates of all-cause death, mostly due to the relatively small sample size. On the other hand, rates of cardiovascular death were lower following LEVO compared to either placebo, furosemide of standard of care in a fixed-effect model meta-analysis. Interestingly, only one study reported that continuous infusion of PGE1 was superior to LEVO regarding a 6-month combined endpoint of death, HTx, or LVAD. These findings require further confirmation in adequately powered study.

### Safety of LEVO in this systematic review

Overall, no major safety concerns regarding LEVO use in ambulatory patients with end-stage HF were reported. However, adverse events associated with repeat infusions of LEVO are not uncommon. The overall rate of such events in ambulatory patients with end-stage HF was not statistically higher than with placebo [7]. It indicates that the peak phase for treatment-related adverse events with LEVO occurs during the infusion itself. It is safer to confine monitoring of adverse events to the infusion period and subsequent 3 h [7]. In contrast to other inotropes such as dobutamine, LEVO, as mentioned above, does not increase intracellular calcium concentration. Its inotropic effect is due to sensitization of the contractile apparatus to calcium ions which is mediated by calcium concentration-dependent conformational changes in troponin-C during systole [25]. Furthermore, vasodilator effect of LEVO is mediated by the activation of ATP-dependent potassium channels in vascular smooth muscle cells [26]. Therefore, systemic hypotension is not uncommon on LEVO. Altenberger et al. reported that LEVO-treated patients were more likely to experience arterial hypotension, although tachycardia and arrhythmia were infrequent and did not differ from placebo [7]. These findings were consistent among several studies.

### Limitations

The small samples, heterogeneity of individual studies in relationship to primary and secondary endpoints, variability in follow-up duration, and protocol of administration of LEVO are important limitations. Furthermore, the pooled data is combining the results from RCTs and observational studies. In addition, some of the outcome measures such as the cost and length of hospitalization was examined in only one or two studies.

## Conclusions

In comparison with conventional HF therapy, intermittent LEVO infusions in ambulatory patients with end-stage HF are safe and are associated with a mitigated HF progression, reflected by reduction in BNP and improved LV size and function alongside with an improved functional status, less frequent hospitalization, shorter hospital stay, and reduced cardiovascular mortality. However, the current evidence of LEVO efficacy and safety is largely based on heterogeneous and relatively small studies. Therefore, a prospective, adequately powered, randomized control trial is highly needed given the very promising results, which can be a game changer for most of the end-stage HF patients in the grave phase of their life, especially if an LVAD implantation or an HTx is not feasible.

## Supplementary Information

Below is the link to the electronic supplementary material.

Supplementary file1 (PDF 561 KB)

## Abbreviations

6MWD: 6 Minute Walk Distance
BNP: B-type natriuretic peptide (BNP)
CMA: Comprehensive meta-analysis
eGFR: Estimated glomerular filtration rate
HF: Heart failure
HRQoL: Heart-related quality of life
HTx: Heart transplantation
KCCQ: Kansas City Cardiomyopathy Questionnaire
LEVO: Levosimendan
LVAD: Left ventricular assist implantation device
LVD-36: Left Ventricular Dysfunction Questionnaire
LVEDV: Left Ventricular End-Diastolic Volume
LVEF: Left ventricular ejection fraction
LVES: Left ventricular end-systolic
LVESV: Left ventricular end-systolic volume
LVFS: Left ventricular fractional shortening
MAP: Mean arterial pressure
MLHFQ: Minnesota Living with Heart Failure Questionnaire
NT-pro-BNP: N-terminal (NT)-pro hormone BNP
NYHA: New York Heart Association
PGE1: Prostaglandin E1
PRISMA: Preferred Reporting Items for Systematic Reviews and Meta-Analysis
SAQ: Specific Activity Questionnaire
WMD: Weighted mean difference
