# Correction to: Intermittent levosimendan infusion in ambulatory patients with end-stage heart failure: a systematic review and meta-analysis of 984 patients

**DOI:** 10.1007/s10741-021-10130-9

**Published:** 2021-06-21

**Authors:** Hagar Elsherbini, Osama Soliman, Casper Zijderhand, Mattie Lenzen, Sanne E. Hoeks, Rasha Kaddoura, Mohamed Izham, Abdulaziz Alkhulaifi, Amr S. Omar, Kadir Caliskan

**Affiliations:** 1 Department of Cardiology, Erasmus University Medical Centre, Rotterdam, Netherlands; 2 Utrecht University of Applied Sciences, Utrecht, Netherlands; 3 Department of Cardiology, National University of Ireland, Galway, Ireland; 4 Department of Clinical Pharmacy, Hamad Medical Corporation, Doha, Qatar; 5 College of Pharmacy, QU Health, Qatar University, Doha, Qatar; 6 Department of Cardiothoracic Surgery/Cardiac Anaesthesia & ICU, Heart Hospital, Hamad Medical Corporation, Doha, Qatar; 7 Department of Critical Care Medicine, Beni Suef University, Beni Suef, Egypt; 8 Department of Clinical Medicine, Weill Cornell Medical College, Doha, Qatar

## Correction to: Heart Fail Rev https://doi.org/10.1007/s10741-021-10101-0

The article “Intermittent levosimendan infusion in ambulatory patients with end‑stage heart failure: a systematic review and meta‑analysis of 984 patients”, written by Hagar Elsherbini, Osama Soliman, Casper Zijderhand, Mattie Lenzen, Sanne E. Hoeks, Rasha Kaddoura, Mohamed Izham5, Abdulaziz Alkhulaifi, Amr S. Omar, and Kadir Caliskan, was originally published electronically on the publisher’s internet portal on 11 April 2021 without open access. With the author(s)’ decision to opt for Open Choice the copyright of the article changed on 10 June 2021 to © The Author(s) 2021 and the article is forthwith distributed under a Creative Commons Attribution 4.0 International License, which permits use, sharing, adaptation, distribution and reproduction in any medium or format, as long as you give appropriate credit to the original author(s) and the source, provide a link to the Creative Commons licence, and indicate if changes were made. The images or other third party material in this article are included in the article’s Creative Commons licence, unless indicated otherwise in a credit line to the material. If material is not included in thearticle’s Creative Commons licence and your intended use is not permitted by statutory regulation or exceeds the permitted use, you will need to obtain permission directly from the copyright holder. To view a copy of this licence, visit http://creativecommons.org/licenses/by/4.0.

The original article has been corrected.

