# Supplementary material for: Intermittent levosimendan infusion in ambulatory patients with end-stage heart failure: a systematic review and meta-analysis of 984 patients
Source: Heart Fail Rev. 2021 Apr 11;27(2):493–505. doi: 10.1007/s10741-021-10101-0 (PMC8898255; doi:10.1007/s10741-021-10101-0)
Supplement: Supplementary file 1 — Supplementary file1 (PDF 561 KB) [file 10741_2021_10101_MOESM1_ESM.pdf]

Intermittent Levosimendan Infusion in Ambulatory Patients with End-Stage Heart Failure. A Systematic Review and Meta-Analysis

Hagar Elsherbini, BSc[a,b], Osama Soliman, MD, PhD[c*], Casper Zijderhand, MD[a], Mattie Lenzen, PhD[a], Sanne E. Hoeks, PhD[a], Rasha Kaddoura, MSc (Pharm), Pharm D[d], Mohamed Izham, Pharm D[e] Abdulaziz Alkhulaifi, FRCS (CTh)[f], Amr S Omar, MD, PhD, MBA[f,g,h] ; and Kadir Caliskan, MD, PhD[a*]

a) Department of Cardiology, Erasmus University Medical Centre, Rotterdam, Netherlands
b) Pharmacy, Utrecht University of Applied Sciences, Utrecht, Netherlands
c) Department of Cardiology, National University of Ireland, Galway, Ireland
d) Department of Clinical pharmacy, Hamad Medical Corporation, Doha, Qatar
e) Faculty of Pharmacy, Qatar University, Doha, Qatar
f) Department of Cardiothoracic Surgery/Cardiac Anaesthesia & ICU, Heart Hospital, [f]Hamad Medical Corporation, Doha, Qatar
g) Department of Critical Care Medicine, Beni Suef University, Egypt
h) Weill Cornell Medical College, Qatar

Table of contents

Pharmacokinetics of Levosimendan 3

Pharmacodynamics of Levosimendan 3

LEVO infusion protocol 4

Safety of Levosimendan 4

Supplementary Table 1. Metadata of of the 15 studies included in the systematic review and meta-analysis 6

Supplementary Table 2A. Methodology of the 15 studies included in the systematic review and meta-analysis – study design. 7

Supplementary Table 2B. Methodology (Endpoints) of the 15 studies included in the systematic review and meta-analysis 8

Supplementary table 3A. Summary of the Levosimendan efficacy in the non-randomized trials 9

Supplementary Table 3B. Summary of the Levosimendan efficacy in the randomized trials 11

Supplementary Table 4. Summary of Levosimendan Safety of the 15 studies included in the systematic review and meta-analysis 13

Supplementary Material Appendix 1 14

Pharmacokinetics of levosimendan

LEVO becomes completely metabolized. Five percent of the intravenously administered LEVO is excreted into the small intestine and reduced in the large intestine by intestinal bacteria to an aminophenolpyridazinone metabolite OR-1855. Thereafter this metabolite will be further metabolized in the liver by acetylation to an active metabolite N-acetylated OR-1896. The elimination time of LEVO is 1-1.5 hours and the total clearance is approximately 200-360 mL/min. Steady state concentrations of LEVO are achieved within 4-8 hours. This can be expected based on the elimination half-life. The metabolite OR-1896 has a half-time of 75 to 80 hours after administration. Due to the half-life of this metabolite, cardiovascular effects persist up for a week after discontinuation of 24-hour of LEVO infusion. Due to the elimination half-life of the metabolites, it can be estimated that steady-state concentration of OR-1855 and OR-1896 could be achieved within two weeks (1,2).

Pharmacodynamics of levosimendan

LEVO causes, an increase in troponin C affinity for calcium, it also presents other effects such as anti-ischaemic effects, cardioprotection and peripheral vasodilator. Due to the mechanism of action of LEVO, myocardial contraction is strengthened without increasing oxygen demand, intracellular calcium concentration at clinically relevant doses or intracellular cAMP.

LEVO binds in the hydrophobic region of the N-domain next to the D/E left region in a calcium dependent manner to the regulatory domain of troponin C. This makes the flexible left region more rigid. This results in the limitation of the freedom degrees of the domains.

LEVO inhibits troponin I effects. LEVO hinders troponin I effects by the stabilizing the conformation of troponin C, due a network of hydrophobic and electrostatic interactions. This mechanism results in deceleration of the dissociation rate and acceleration of actin myosin

crossbridge formation rate. The moment the calcium intracellular level is low, is the binding weaker during diastole. This leads to positive inotropic properties, such as myocardial contractility, relaxation, and improvement in LV diastolic function, without increasing myocardial oxygen demand or impairing ventricular relaxation (2).

LEVO infusion protocol

In 10 (3-12) out of 15 studies (3-17), no loading dose of LEVO was used. Initial intravenous bolus of 6 ug/kg *over* 10-minute was used in 3 studies. Intravenous bolus of 6 ug/kg over 10-minute following 24 h duration of dobutamine was used in 1 study. Only in one study, intravenous bolus of 12 ug/kg over 10-minute was used provided that SBP ≥95 mmHg. Following the bolus, if applicable, a maintenance dose was used (range 0.10 to 0.40 μg/kg/min for 24h up to a maximum of 12.5mg per session). Duration of infusion per cycle ranges from 6 to 48hrs. Number of cycles ranged from 1 to 26 cycles per patient per study. Frequency of infusion ranged from weekly to bi-monthly. *Table 2B* lists all detailed infusion protocol in the 15 studies.

Safety of Levosimendan

Adverse events of intermittent LEVO infusion were reported in 10 studies. Hypotension was reported in 2.2% in Oliva et al.(10), in 36% in Ortis et al. (11), in 8% in Parle et al.(16), in 20% in Tasal et al. (17), in 8.9% in Altenberger et al.(3), and in 4% in Mavrogeni et al.(8). Both Berger et al. (13) and Comín-Colet et al. (5) reported that the frequency of hypotension was not different between LEVO and PGE1 or placebo, respectively.

Asymptomatic non-sustained ventricular tachycardia was observed during 4(2.5%) infusions (16), ventricular arrhythmias in 16(8.6%)(10) and atrial fibrillation in 1(0.5%)(10). Arrhythmia rates were similar between LEVO and placebo in 3 studies(3,7,8), and versus furosemide in Malfatto et al. Amiodarone 400mg OD was used to mitigate the proarrhythmic effects of

inotropic agents in 2 studies(4,6) with no adverse events related to LEVO infusion. Detailed of Levosimendan safety are presented on *Supplementary file and supplementary Table3C.*

A total of 8 studies(3,5,8,10,11,13,16,17) reported hemodynamic instability (hypotension or unstable arrhythmias) associated with LEVO (*Supplementary Tables 3B and Table 3C*). Altenberger et al.(3) reported a drop in SBP on LEVO versus placebo (-6.7mmHg, [IQR -16.6 to -2.7mmHg] versus -1.0mmHg, [IQR-4.5 to +3.5mmHg]; p=0.01). Vasopressor use or fluid administration for symptomatic hypotension were used in 8.9% of patients in both groups. Comin-Colet et al.(5) found no difference in SBP or arrhythmia between LEVO and placebo.

Arterial hypotension was reported in 7 studies. Berger et al.(13) found no difference in arterial hypotension between LEVO and PGE1. Hypotension caused LEVO dose reduction in 12(16%) patients and inability to start repeated dose in 3(4%) patients (13). Mavrogeni et al.(8) reported dose reduction due to hypotension in 2 patients but tolerated later maximum LEVO dose. Systemic hypotension was seen ~~in~~ in 4(2.2%) patients in Oliva et al.(10) and in 4(2.2%) patients following LEVO in Ortis et al.(11). Parle et al.(16) reported that 83.5% of infusions were completed without adverse events. Symptomatic hypotension was seen in 8% infusions with a drop of mean SBP to 41.7(6.4) mmHg. Seven episodes of hypotension resolved with dose reduction, 3 episodes required noradrenaline and 2 infusions were discontinued prematurely. Tasal et al.(17) reported 11(20%) LEVO infusions was shortly interrupted due to hypotension but later, all patients completed infusion without any adverse events.

Supplementary Table 1. Metadata of of the 15 studies included in the systematic review and meta-analysis

Author	Date of Publication	Journal	Acronym	Database	DOI/PMID
**Altenberger et al.**	2014	European Journal of Heart Failure	LevoRep	Embase	doi:10.1002/ejhf.118
**Berger et al.**	2006	The European Journal of Heart Failure	NA	Embase	doi:10.1016/j.ejheart.2006.06.001
**Bonios et al.**	2011	International journal of cardiology	NA	Embase	doi:10.1016/j.ijcard.2011.03.013.
**Comín-Colet et al.**	2018	European Journal of Heart Failure	LION-HEART	Embase	doi:10.1002/ejhf.1145
**Drakos et al.**	2008	Journal of cardiovascular pharmacology	NA	Embase	doi:10.1097/FJC.0b013e31819846cd
**Malfatto et al.**	2012	Journal of Cardiovascular Pharmacology	NA	Embase	doi:10.1097/FJC.0b013e31826b86aa
**Mavrogeni et al.**	2007	Journal of Cardiac Failure	NA	Embase	doi:10.1016/j.cardfail.2007.04.004
**Najjar et al.**	2018	ESC Heart failure	NA	Embase	doi: 10.1002/ehf2.12272
**Nanas et al.**	2015	The American Journal of Cardiology	NA	Embase	doi:10.1016/j.amjcard.2004.11.033
**Oliva et al.**	2018	International journal of cardiology	NA	Embase	https://doi.org/10.1016/j.ijcard.2018.08.048
**Ortis et al.**	2016	Journal of International Medical Research	NA	Embase	doi: 10.1177/0300060516655244
**Papadopoulou et al.**	2009	Hellenic J Cardiol	NA	Embase	PMID: 19622496
**Parissis et al.**	2006	Cardiovascular Medicine	NA	Embase	doi: 10.1136/hrt.2006.079707
**Parle et al.**	2008	Heart, Lung and Circulation	NA	Embase	doi:10.1016/j.hlc.2007.10.014
**Tasal et al.**	2014	Medical science monitor	NA	Embase	doi: 10.12659/MSM.889767

Abbreviations: NA=Not Available

Supplementary Table 2A. Methodology of the 15 studies included in the systematic review and meta-analysis – study design.

Author	Study design and patients	Randomized (yes, No)
**Altenberger et al. 2014**	Prospective, randomized, double-blind, placebo-controlled, multicentre, parallel-group	Yes
**Berger et al. 2006**	Prospective, randomised, open, parallel group	Yes
**Bonios et al. 2011**	Randomized, open-labelled study	Yes
**Comín-Colet et al. 2018**	Multicentre, double-blind, randomised, parallel-group, placebo-controlled	Yes
**Drakos et al. 2008**	Prospective study	No
**Malfatto et al. 2012**	Randomized open-label PRO patient-reported outcomes	Yes
**Mavrogeni et al. 2007**	Open, prospective, double-blind randomised controlled trial	Yes
**Najjar et al. 2018**	Prospective single-centre single-arm	No
**Nanas et al. 2015**	Prospective, non-randomised, sequentially comparing two treatment strategies (dobutamine vs dobutamine + levosimendan)	No
**Oliva et al. 2018**	Prospective multicentre registry	No
**Ortis et al. 2016**	Retrospective study compared with controls	No
**Papadopoulou et al. 2009**	Prospective	No
**Parissis et al. 2006**	Randomised placebo-controlled	Yes
**Parle et al. 2008**	Prospective, single-centre experience	No
**Tasal et al. 2014**	Non-randomised, non-controlled	No

Supplementary Table 2B. Methodology (Endpoints) of the 15 studies included in the systematic review and meta-analysis

Author	Primary endpoint(s)	Secondary endpoints
Altenberger et al. 2014	• >20% increase in 6MWD and<br>• >15% increase in KCCQ	• Event-free survival<br>• 6MWT<br>• KCCQ<br>• NT-pro-BNP levels >30% drop
Berger et al. 2006	• Worsening of HF	• A combined endpoint of death, urgent heart transplantation or implantation of a ventricular assist device (VAD).
Bonios et al. 2011	• Death or urgent LVAD at 3&6 months.	• Death, elective** or urgent *LVAD implantation or hospitalization for HF decompensation
Comín-Colet et al. 2018	• NTproBNP	• Safety, clinical and HRQoL<br>• Clinical events (hospitalization, death and other terminal events such as HTx, LVAD)
Drakos et al. 2008	• Death from any cause	• Changes in functional status, hemodynamic variables, LVEF, left ventricular dimensions, and maximum oxygen uptake
Malfatto et al. 2012	• NYHA-class	• BNP
Mavrogeni et al. 2007	• SAQ<br>• LVEF	• Improvement in Symptoms<br>• LV shortening fraction<br>• Mitral regurgitation
Najjar et al. 2018	• Cardiac output (CO)<br>• NT-pro-BNP	• MAP
Nanas et al. 2015	• 45-day survival rates<br>• LVEF, NYHA, CI, PCWP, SVR	
Oliva et al. 2018	• Days In hospital ratio (DIH%, 6m before / 6m after LEVO)	• Number&length of hospitalizations<br>• A combination of death/urgent HTx/LVAD implant at 12m<br>• Cost<br>• Need to decrease infusion dose, rate or interval between infusions
Ortis et al. 2016	• Mortality (12M)<br>• Hospitalizations (6M, 12M)<br>• length of hospital stay	• Clinical<br>• Lab<br>• Echo (LVEF)
Papadopoulou et al. 2009	• SAQ<br>• LVD 36<br>• MLIhFE	• LVEF
Parissis et al. 2006	• LVEF<br>• LVES wall stress	• NT-proBNP
Parle et al. 2008	• NYHA-class	• BNP
Tasal et al. 2014	• Changes in clinical, lab & echo after 1 dose & at 6m	• BNP<br>• IL-6<br>• TNF-α

Abbreviations: IV=intravenous; LEV=Levosimendan; PL=Placebo; DOB= dobutamine; LVAD= Left ventricular assist device; LVEF=Left ventricular ejection fraction; MOA=Method of administration; BNP= Brain natriuretic peptide levels; SAQ=Specific Activity Questionnaire; LVD36=LV Dysfunction 36; MLIhFE=Minnesota Living with Heart failure Questionnaire; VAD= implantation of a ventricular assist device; PRO=patient-reported outcomes; NR=Not reported; CI=Cardiac index; PCWP=Pulmonary capillary wedge pressure; SVR=Systemic vascular resistance *Urgent HTx= urgent (i.e. unscheduled previously, following an episode of decompensation with the patient hospitalized. ** Elective = scheduled, with the patient ambulatory and followed as an outpatient, NR= Not Reported

Supplementary table 3A. Summary of the Levosimendan efficacy in the non-randomized trials

Author	Primary endpoint outcome	Main Secondary endpoint	Overall efficacy of LEVO
**Drakos et al.**	• **Survival at 6 months** Inotropes agents (DOB+LEVO) vs control (51% vs 18%, P=0.001)<br>• **Survival at 12 months** Inotropic agents (DOB+LEVO) vs control (36% vs 9%, P=0.001)<br>• **The median survival time:** Inotropic agents (DOB+LEVO) vs control (182 vs 84 days, Kaplan-Meier survival estimates log-rank, P<0.001).	• **LVEF** Inotropic agents baseline vs 6-months (23.6±7.3 vs 27.7±9.9, P=0.04)<br>• **Heart rate** Inotropic agents baseline vs 6-months (76±14 vs 71±11, P=0.036)<br>• **NYHA** Inotropic agents baseline vs 6-months (4±0 vs 2.8±0.8, P=0.0001)	**Primary endpoint:**<br>• Positive for survival for inotropes<br>**Secondary endpoint:**<br>• Positive for LVEF and NYHA and<br>• Peutral effect for Heart rate
**Najjar et al.**	**Pre- vs post-LEVO**<br>• **Cardiac output** (3.05 vs 3.45 L/min, p=0.03)<br>• **NT-pro-BNP** (3400 vs 2530 pg/mL, P<0.001)	**Pre- vs post-LEVO**<br>• MAP (79 vs 74 mmHg, P=0.007)<br>• eTPR (1628 vs 1343 dync.s/cm^5, P=0.004)<br>• eGFR (62 vs 61 mL/min/m^2, P=0.96)	**Outcome measures**<br>• CO, NT-pro-BNP, MAP and eTPR are all positive for LEVO
**Oliva et al.**	**The 6m after start vs the 6m before LEVO**<br>• Days in hospital (DIH%): 9.4(8.2%) versus 2.8(6.6%), (p<0.0001)	**The 6m after start vs the 6m before LEVO**<br>• Hospitalization<br>○ Number (1.3 vs 1.8 days, P 0.0001)<br>○ Length (17.4 vs 21.6 days, p=0.0001)<br>• Direct costs Lower by 1157(€8676), p=0.05.	**Primary endpoint:**<br>• Hospitalization ratio: positive<br>**Secondary: endpoint:**<br>• Hospitalization no: positive<br>• Hospitalization length: positive<br>• Costs: NS
**Ortis et al.**	**(LEVO vs Control)**<br>• **Mortality:** 3(12%) vs 2(8%), p=NS<br>• **No. of hospitalizations/patient** Baseline: 0.84±0.80 vs 0.40±0.70, P<0.05<br>6m: 0.24±0.52 vs 0.83±1.02, P<0.05<br>12m: 0.68±0.80 vs 1.72±1.42, P<0.05<br>• **Length of stay in hospital/patient, days** Baseline 9.2±13.1 vs 2.5±5.1, p<0.05<br>a6m: 1.8±4.7 vs 8.0±14.1, p<0.05<br>12m: 8.9±17.0 vs 15.9±19.9, p<0.05)	**Baseline vs 12 months**<br>• **LVEF LEVO**<br>(26.8 ± 5.6 vs 28.8 ± 6.5, p=0.05)<br>• **LVEF Control**<br>(33.1 ± 8.9 vs 33.0 ± 9.6, p=NS)<br>• **BNP LEVO**<br>(900.4 ± 380.5 vs 872.3 ± 605.3, p=NS)<br>• **BNP Control**<br>(655.7 ± 513.9 vs 807.1 ± 755.4, p= 0.046)	**Primary endpoints**<br>• Mortality: similar<br>• Hospitalization: LEVO is better at all timepoints and in comparison, with baseline<br>**Secondary endpoints**<br>• LVEF is positive for LEVO<br>• BNP neutral for LEVO, worsened for Control
**Papadopoulou et al.**	**Before vs 6-months post-LEVO**	**Before vs 6-months post-LEVO**	**Outcome measures**<br>• Positive for all 3 questionnaires

Author	Primary endpoint outcome	Main Secondary endpoint	Overall efficacy of LEVO
	• SAQ (4.2±1.6% vs 4.7±1.3%, p<0.05)<br>• LVD36 (52.6±26.2 vs 27.4±17.3, p<0.0001)<br>• LIhFE (35.4±18.6 vs 22.2±13.0, p<0.0001)	• Mean LVEF (30.3± 6.9 vs 32.1±7.4, p=0.01)	• Positive for mean LVEF for LEVO
**Parle et al.**	• NYHA-class IV<br>Pre- vs post infusion (58%) vs (19%), p<0.0001)	• BNP levels (Pre- vs post-infusion)<br>(1081.1 (176.0) vs 703.3 (84.8) ng/L, P <0.01)<br>• MAP (Pre- vs post-infusion) (74.4 (0.8) vs 74.0 (0.7) mmHg, p=NS)	**Outcome measures**<br>• Positive for NYHA class<br>• Positive for BNP<br>• Neutral effect on MAP
**Tasal et al.**	**6m single dose (pre- vs post-infusion)**<br>• **LVESV** (155.8±40.0 vs 155.5± 40, p<0.05)<br>• **LVEDV** (212.5±34.9 vs 212.6±35.4, p=NS)<br>• **MPI** (1.08 ± 0.19 vs 1.07 ± 0.18, p<0.05)<br>• **SM** (8.3 ± 0.8 vs 8.2 ± 1.0, p<0.05)<br>• **LVEF** (29.7 ± 2.7 vs 29.4 ± 3.1, p<0.05)<br><br>**6m repeated dose (pre- vs post-infusion)**<br>• LVESV (159.3±43.1 vs 138± 40.2, p<0.05<br>• LVEDV (221.9±58.8 vs 216.8±38.3, p<0.05)<br>• MPI (1.11 ± 0.2 vs 0.92 ± 0.1, P<0.05)<br>• SM (8.03 ± 1.0 vs 9.6 ± 1.01, P<0.05)<br>• LVEF (29.1 ± 3.3 vs 33.7 ± 3.4, P<0.05)	**6m single dose (pre- vs post-infusion)**<br>• **BNP** (2015.3 ±871.3 vs 1811.0±654.7, p<0.05)<br>• **IL-6** (14.6 ± 9.8 vs 16.33 ± 12.8, p<0.05)<br>• **TNF-α** (6.1± 3.9 vs 6.4 ± 3.3, p<0.05)<br><br>**6m repeated dose (pre- vs post-infusion)**<br>• **BNP** (2306.4± 775.1 vs 891.8 ± 438.9, p<0.05)<br>• **IL-6** (24.8 ± 22.2 vs 5.5 ± 4.1, p<0.05)<br>• **TNF-α** (7.8± 4.1 vs 5.2 ± 3.7, p<0.05)<br><br>**Single- vs. repeated-dose**<br>• NYHA-class baseline<br>(3.8 ± 0.4 vs. 3.7 ± 0.4, p=0.5)<br>• NYHA-class Follow-up<br>(1.5 ± 0.7 vs 0.9 ± 0.6, p=0.03)	**Outcomes measures**<br>• All positive except LVEDV for single-dose of LEVO, but positive for repeated-dose of LEVO<br>• All positive except NYHA class. NYHA class neutral for single-dose vs repeated dose for LEVO prior treatment, but positive at 6 months of follow-up

Abbreviations: Supplementary Table 2B

Supplementary Table 3B. Summary of the Levosimendan efficacy in the randomized trials

Author	Primary endpoint outcome	Main Secondary endpoint	Overall efficacy of LEVO
**Altenberger et al.**	**>20% increase in 6MWT and >15% score increase in KCCQ**<br>In LEVO vs PLA at 8 wks: (23.8% vs 21.1%, p=0.82); and at 24 wks (19% vs 15.8%, p=0.81), respectively.	• **Event-free survival**<br>LEVO vs Placebo (82.6% vs 64.9%, p=0.04)<br>• **Event rate (death, urgent HTx, AHF)**<br>LEVO vs PLA (17.4% vs 35.1%, p=0.04)<br>• **Kaplan-Meier (24 weeks)**<br>(HR 0.50, 95% CI 0.24–1.025; p=0.07)<br>• **NT-pro-BNP>30% drop at 8 wks:** LEVO vs PLA (31.7% vs 14%, p=0.03)<br>• **NT-pro-BNP>30% drop at 24 wks:**<br>LEVO vs PLA (17.5% vs 10.5%, p=0.3)	**Primary endpoint:**<br>• No significant difference<br>**Secondary endpoints:**<br>• Event-free survival after 24 wks (positive for LEVO)<br>• NT-proBNP drop >30%<br>• Positive for LEVO at 8 wks<br>• Negative for LEVO at 24 wks
**Berger et al.**	• **Worsening of heart failure**<br>LEVO vs PGE1 (74% vs 44%, p=0.008)	• **Individual endpoints: LEVO vs PGE1**<br>LVAD (18% vs 6%, p=0.09)<br>HTx (8% vs 0, p=0.09)<br>Death (5% vs 6%, p=NS)<br>• **Combined endpoint of death or HTx or LVAD**<br>At 12M (31% vs 11%, p=0.04)<br>• In both groups LVEF significantly increased by 5 to 6% points, and the NYHA<br>improved by 1.4 classes.<br>• Taking both study groups together, BNP levels decreased significantly.	**LEVO vs PGE1**<br><br>**Primary endpoint:**<br>• Negative for LEVO vs PGE1 at 3m<br>• Similar at 1-year<br>**Secondary endpoint:**<br>• All negative for LEVO vs PGE1 except death (neutral) at 3m<br>• Similar at 1-year
**Bonios et al.**	• **3M Survival free from death or urgent LVAD**: LEVO= 90% vs DOB= 62%<br>p=0.047) DOB + LEVO= 51%(p=0.006)<br>• **Survival free from death or urgent LVAD at 6 months**<br>LEVO= 80% DOB= 48% (p=0.037) DOB+LEVO= 43% (p=0.009)	• **The 6M survival free from death, urgent LVAD or hospitalization for HF deterioration**<br>LEVO= 44%<br>DOB=44%<br>LEVO+DOB= 30%<br>(p=NS)	**Primary endpoint:**<br>• Positive for LEVO<br>**Secondary endpoint:**<br>• Neutral for LEVO
**Comín-Colet et al.**	• **NT-proBNP (AUC pg.day/mL) LEVO vs PLA**<br>[344 × 10^3, 95% CI 283 × 10^3 –404 × 10^3<br>Vs.<br>535 × 10^3, 95% CI 443 × 10^3 – 626 × 10^3; p = 0.003)	**LEVO vs PLA**<br>• **HTx, LVAD or death** (31% vs 48%, p=0.30)<br>• **All-cause death** (31.2 % vs 38.1%, p=0.78)<br>• **All-cause hospitalization** (35.4% vs 71.4%, p=0.01)<br>• **CV hospitalization or HTx or LVAD or death**<br>(45.8% vs 81.0%, p=0.02)<br>• **NYHA** (>1 NYHA class improvement [OR 4.3, 95% CI 1.1–18.3; p=0.04].<br>• **HRQoL**(less clinically significant decline (EQ-5D VAS [5/24 (21%) vs. 7/11 (63%), p=0.02]	**Primary endpoint (12 wks):**<br>• Positive for BNP for LEVO<br>**Secondary endpoints (6 months):**<br>• Death LEVO similar to PLA<br>• Positive for composite for LEVO<br>• Positive for hospitalization for LEVO<br>• Positive for NYHA for LEVO<br>• Positive for QoL for LEVO<br>• Adverse events similar to PLA
**Malfatto et al.**	• **NYHA-class (LEVO)**<br>**Baseline vs LEVO after the**	• **BNP**<br>Baseline vs LEVO after the first infusion	**Outcome measures**<br>• Positive for LEVO

Author	Primary endpoint outcome	Main Secondary endpoint	Overall efficacy of LEVO
	**first infusion**<br>(3.07 ± 0.36 vs 2.55 ± 0.33, p<0.01)<br>Baseline vs LEVO after 4th infusion<br>(3.07±0.36 vs 2.45 ± 0.23, p<0.01)<br>• **NYHA-class (Furosemide vs control)**<br>At baseline vs first visit (3.16±0.39 vs 3.09±0.54, (p<0.05)<br>At baseline vs at the time of 4th infusion (3.16±0.39 vs 3.09±0.54, (p<0.05)	(1033±807 vs 338±249, P<0.001)<br>Baseline vs LEVO after 4th infusion (1033±807 vs 362±210, p<0.001)<br>• **Severe FMR**<br>Baseline vs LEVO after the first infusion (7/22 (32%) vs 1/22 (5%), p<0.05)<br>Baseline vs LEVO after 4th infusion (7/22 (32%) vs 0/22 (0%), p<0.05)	• Positive for furosemide<br>• Positive for LEVO<br>• Positive for furosemide
**Mavrogeni et al.**	• **SAQ (LEVO vs control after 6 months)**<br>(2.7 ± 1.2 vs 1.7 ± 1.6, p=0.42)<br>• **LVEF (LEVO vs control after 6 months)**<br>(28 ± 7 vs 22 ± 6, p=0.003)	**LEVO vs Control**<br>• **Improved in symptoms** (65% vs 20%, p<0.01)<br>• **LVFS** (15±3 vs 11±3, p=0.006)<br>• **MR grade** (1.5±0.8 vs 2.7±0.6, p=0.0001)<br>• **LVEDV** (120±20 vs 157±25, p=0.0001)<br>• **LVESV** (8 ±20 vs 107±21, p=0.04)	**Outcome measures (LEVO)**<br>• Positive for LVEF, LVFS, LVEDV, MR grade severity<br>• Positive for symptoms<br>• Negative for SAQ
**Nanas et al.**	• **45-day survival rates**<br>DOB + LEVO vs DOB only (61% and 6%, respectively; p<0.0002, log-rank test)	**(LEVO baseline vs 3 months)**<br>• **LVEF** (18 ± 5 vs 21 ± 4, p= 0.04)<br>• **NYHA** (4±0 vs 2.2±0.4, p=0.001)<br>• **PCWP** (31±7 vs 14±7, p=0.03)<br>• **CI** (1.41±0.20 vs 2.39±0.30, p=0.0001)<br>• **SVR** (23±5 vs 16±3, p=0.007)	**Outcome measures**<br>• Survival is positive for LEVO<br>• LVEF, PCWP, CI, SVR and NYHA are all positive for LEVO
**Parissis et al.**	**Baseline vs final**<br>• **LVEDVI**<br>LEVO (133±25 vs 120±28, p<0.05)<br>PLA (144±29 vs 156±30, p<0.05)<br>• **LVESVI**<br>LEVO (95±28 vs 80±25 p<0.05)<br>PLA (98±25 vs 106±22, p<0.05)<br>• **LVEF**<br>LEVO (22% vs 26%, p<0.01)<br>PLA (23% vs 22%, p=NS)<br>• **LVES wall stress**<br>LEVO (859 vs 748, p<0.01)<br>PLA (807 vs 828, p<0.05)	**Baseline vs final**<br>• **NT-pro-BNP**<br>LEVO (1547 vs 966, p<0.01)<br>PLA (1302 vs 1529, p<0.01)<br>• **Troponin T**<br>LEVO (5/12 vs 6/11, p=NS)<br>PLA (3/5 vs 1/7, p<0.05)<br>• **Inflammatory markers**<br>LEVO (HsCRP and IL-6, sig reduced)<br>PLA (no reduction)	**Outcome measures:**<br>• Positive for LV size reduction for LEVO vs worsened LV size on PLA<br>• Positive for LVEF for LEVO<br>• Positive (LVES wall stress was reduced on LEVO and increased on PLA)<br>• Positive for NT-pro-BNP<br>• Neutral effect for LEVO on Troponin T<br>• Worsened Troponin T on PLA

Abbreviations: LVFS=Left ventricular shortening fraction; MR grade=Mitral regurgitation; EDV=End-diastolic volume; ESV=End-systolic volume; MAP=Mean arterial pressure; FMR=Functional mitral regurgitation; LVESV=Left ventricular end systolic volume; LVEDV=Left ventricular end diastolic volume; SM=Peak systolic tissue Doppler velocity at lateral corner of mitral annulus; LVEF=Left ventricular ejection fraction; BNP= B type natriuretic peptide; IL-1β= Interleukin-1 beta; TNF-α=Tumour necrosis factor-alfa; IL-6= Interleukin 6; IL-2= Interleukin -2;NYHA= New York heart association.

Supplementary Table 4. Summary of Levosimendan Safety of the 15 studies included in the systematic review and meta-analysis

Author	Overall safety of LEVO	Arterial hypotension	Arrhythmias
**Table 3C. Summary of the Levosimendan Safety in the Non-Randomized trials (N=7)**			
**Drakos et al.**	No significant change in heart rate		All patients received amiodarone 400 mg OD to mitigate the proarrhythmic effects of inotropic agents
**Najjar et al.**	• Adverse events was not reported<br>• eGFR is stable on LEVO		
**Oliva et al.**	• Worsening renal function 4(2.2%)	Hypotension 4 (2.2%)	• Ventricular arrhythmias 16(8.6%)<br>• Atrial fibrillation 1 (0.5%)
**Ortis et al.**		Asymptomatic hypotension requiring lowering infusion rate in 9 (36%).	
**Papadopoulou et al.**	• Adverse events was not reported		
**Parle et al.**	• 83.5% infusions completed without any adverse events.	Symptomatic hypotension was seen in 8% infusions	Asymptomatic NSVT was during 4(2.5%) infusions
**Tasal et al.**		LEVO infusion was shortly interrupted 11 times (20%) during infusion due to of hypotension.	No atrial fibrillation, ventricular tachycardia, or fibrillation developed, and no patient died.
Supplementary Table 3D. **Summary of the Levosimendan Safety in the Randomized trials (n=8)**			
**Altenberger et al.**		Active measures for symptomatic hypotension were reported in 8.9% of patients in both groups (p=NS).	Tachycardia was similar between groups
**Berger et al.**		Frequency of hypotension (SBP<90 mm Hg) was not different between LEVO and PGE1.	
**Bonios et al.**	Adverse events was not reported		All patients received amiodarone 400 mg OD to mitigate the proarrhythmic effects of inotropic agents
**Comín-Colet et al.**		Numerically, more patients needed reduction or discontinuation of the infusion due to hypotension in the Levosimendan than in the Placebo group (not statistically significant).	
**Malfatto et al.**			No increase in arrhythmias in LEVO vs. furosemide
**Mavrogeni et al.**		Hypotension in only 2 patients a temporary dose limiting event.	No increase in arrhythmias in LEVO group, compared with controls.
**Nanas et al.**	• Adverse events was not reported		
**Parissis et al.**	• Adverse events were not reported<br>• No significant increase in Troponin T		

Supplementary Material Appendix 1

Search Strategy (terms)Database	Items
**Embase.com**	236
**Medline Ovid**	174
**Web of Science Core Collection**	32
**Cochrane CENTRAL register of trials**	22
**Google scholar**	50
**Total**	**514**

Embase.com 236

('levosimendan'/de OR 'inotropic agent'/mj OR 'cardiotonic agent'/mj OR (levosimendan OR Simendan):ab,ti OR (inotrop* OR cardiotropic* OR cardiotonic*):ti) AND ('end stage heart failure'/de OR 'heart failure'/exp OR (((heart OR cardiac OR myocard*) NEAR/3 fail*) OR hf):ab,ti) AND ('ambulatory care'/de OR 'home care'/exp OR 'outpatient'/de OR 'outpatient care'/de OR 'home infusion therapy'/de OR (ambula* OR outpatient* OR homecare OR home):ab,ti)

Medline Ovid 174

(Simendan/ OR * Cardiotonic Agents/ OR (levosimendan OR Simendan).ab,ti. OR (inotrop* OR cardiotropic* OR cardiotonic*).ti.) AND (exp Heart Failure/ OR (((heart OR cardiac OR myocard*) ADJ3 fail*) OR hf).ab,ti.) AND (exp Ambulatory Care/ OR exp Home Care Services/ OR Outpatients/ OR Home Infusion Therapy/ OR (ambula* OR outpatient* OR homecare OR home).ab,ti.)

Web of Science Core Collection 32

TS=(((levosimendan OR Simendan) OR (inotrop* OR cardiotropic* OR cardiotonic*):ti) AND ((((heart OR cardiac OR myocard*) NEAR/2 fail*) OR hf)) AND ((ambula* OR outpatient* OR homecare OR home)))

Cochrane CENTRAL register of trials 22

((levosimendan OR Simendan):ab,ti OR (inotrop* OR cardiotropic* OR cardiotonic*):ti) AND ((((heart OR cardiac OR myocard*) NEAR/3 fail*) OR hf):ab,ti) AND ((ambula* OR outpatient* OR homecare OR home):ab,ti)

Google scholar 50

levosimendan|Simendan "heart|cardiac|myocard failure" ambulatory|outpatient|homecare|home

References

1. Antila S, Sundberg S, Lehtonen LA. Clinical pharmacology of levosimendan. Clin Pharmacokinet 2007;46:535-52.
2. Pathak A, Lebrin M, Vaccaro A, Senard JM, Despas F. Pharmacology of levosimendan: inotropic, vasodilatory and cardioprotective effects. J Clin Pharm Ther 2013;38:341-9.
3. Altenberger J, Parissis JT, Costard-Jaeckle A et al. Efficacy and safety of the pulsed infusions of levosimendan in outpatients with advanced heart failure (LevoRep) study: a multicentre randomized trial. Eur J Heart Fail 2014;16:898-906.
4. Bonios MJ, Terrovitis JV, Drakos SG et al. Comparison of three different regimens of intermittent inotrope infusions for end stage heart failure. Int J Cardiol 2012;159:225-9.
5. Comin-Colet J, Manito N, Segovia-Cubero J et al. Efficacy and safety of intermittent intravenous outpatient administration of levosimendan in patients with advanced heart failure: the LION-HEART multicentre randomised trial. Eur J Heart Fail 2018;20:1128-1136.
6. Drakos SG, Kanakakis JV, Nanas S et al. Intermittent inotropic infusions combined with prophylactic oral amiodarone for patients with decompensated end-stage heart failure. J Cardiovasc Pharmacol 2009;53:157-61.
7. Malfatto G, Della Rosa F, Villani A et al. Intermittent levosimendan infusions in advanced heart failure: favourable effects on left ventricular function, neurohormonal balance, and one-year survival. J Cardiovasc Pharmacol 2012;60:450-5.

8. Mavrogeni S, Giamouzis G, Papadopoulou E et al. A 6-month follow-up of intermittent levosimendan administration effect on systolic function, specific activity questionnaire, and arrhythmia in advanced heart failure. J Card Fail 2007;13:556-9.
9. Najjar E, Stalhberg M, Hage C et al. Haemodynamic effects of levosimendan in advanced but stable chronic heart failure. ESC Heart Fail 2018;5:302-308.
10. Oliva F, Perna E, Marini M et al. Scheduled intermittent inotropes for Ambulatory Advanced Heart Failure. The RELEVANT-HF multicentre collaboration. Int J Cardiol 2018;272:255-259.
11. Ortis B, Villani A, Oldani M et al. Intermittent levosimendan infusions in advanced heart failure: a real world experience. J Int Med Res 2017;45:361-371.
12. Papadopoulou EF, Mavrogeni SI, Dritsas A, Cokkinos DV. Assessment of quality of life using three activity questionnaires in heart failure patients after monthly, intermittent administration of levosimendan during a six-month period. Hellenic J Cardiol 2009;50:269-74.
13. Berger R, Moertl D, Huelsmann M et al. Levosimendan and prostaglandin E1 for uptitration of beta-blockade in patients with refractory, advanced chronic heart failure. Eur J Heart Fail 2007;9:202-8.
14. Nanas JN, Papazoglou P, Tsagalou EP et al. Efficacy and safety of intermittent, long-term, concomitant dobutamine and levosimendan infusions in severe heart failure refractory to dobutamine alone. Am J Cardiol 2005;95:768-71.
15. Parissis JT, Adamopoulos S, Farmakis D et al. Effects of serial levosimendan infusions on left ventricular performance and plasma biomarkers of myocardial injury and neurohormonal and immune activation in patients with advanced heart failure. Heart 2006;92:1768-72.

16. Parle NM, Thomas MD, Dembo L, Best M, Driscoll GO. Repeated infusions of levosimendan: well tolerated and improves functional capacity in decompensated heart failure - a single-centre experience. Heart Lung Circ 2008;17:206-10.

17. Tasal A, Demir M, Kanadasi M et al. Comparison of single-dose and repeated levosimendan infusion in patients with acute exacerbation of advanced heart failure. Med Sci Monit 2014;20:276-82.
